# Genome-wide association study on legendre random regression coefficients for the growth and feed intake trajectory on Duroc Boars

**DOI:** 10.1186/s12863-015-0218-8

**Published:** 2015-05-30

**Authors:** Jeremy T. Howard, Shihui Jiao, Francesco Tiezzi, Yijian Huang, Kent A. Gray, Christian Maltecca

**Affiliations:** Department of Animal Science, North Carolina State University, Raleigh, NC 27695-7627 USA; Smithfield Premium Genetics, Rose Hill, NC 28458 USA

**Keywords:** Swine, Random regression, Genome wide-association study, Growth and feed intake

## Abstract

**Background:**

Feed intake and growth are economically important traits in swine production. Previous genome wide association studies (GWAS) have utilized average daily gain or daily feed intake to identify regions that impact growth and feed intake across time. The use of longitudinal models in GWAS studies, such as random regression, allows for SNPs having a heterogeneous effect across the trajectory to be characterized. The objective of this study is therefore to conduct a single step GWAS (ssGWAS) on the animal polynomial coefficients for feed intake and growth.

**Results:**

Corrected daily feed intake (**DFI**_**Adj**_) and average daily weight measurements (**DBW**_**Avg**_) on 8981 (n = 525,240 observations) and 5643 (n = 283,607 observations) animals were utilized in a random regression model using Legendre polynomials (order = 2) and a relationship matrix that included genotyped and un-genotyped animals. A ssGWAS was conducted on the animal polynomials coefficients (intercept, linear and quadratic) for animals with genotypes (DFI_Adj_: n = 855; DBW_Avg_: n = 590). Regions were characterized based on the variance of 10-SNP sliding windows GEBV (**WGEBV)**. A bootstrap analysis (n =1000) was conducted to declare significance. Heritability estimates for the traits trajectory ranged from 0.34-0.52 to 0.07-0.23 for DBW_Avg_ and DFI_Adj_, respectively. Genetic correlations across age classes were large and positive for both DBW_Avg_ and DFI_Adj_, albeit age classes at the beginning had a small to moderate genetic correlation with age classes towards the end of the trajectory for both traits. The WGEBV variance explained by significant regions (P < 0.001) for each polynomial coefficient ranged from 0.2-0.9 to 0.3-1.01 % for DBW_Avg_ and DFI_Adj_, respectively. The WGEBV variance explained by significant regions for the trajectory was 1.54 and 1.95 % for DBW_Avg_ and DFI_Adj_. Both traits identified candidate genes with functions related to metabolite and energy homeostasis, glucose and insulin signaling and behavior.

**Conclusions:**

We have identified regions of the genome that have an impact on the intercept, linear and quadratic terms for DBW_Avg_ and DFI_Adj_. These results provide preliminary evidence that individual growth and feed intake trajectories are impacted by different regions of the genome at different times.

**Electronic supplementary material:**

The online version of this article (doi:10.1186/s12863-015-0218-8) contains supplementary material, which is available to authorized users.

## Background

The use of genomic information to infer the estimated breeding value (EBV) of an individual, referred to as genomic-EBV (**GEBV**), has become a routine practice in several livestock species due to the rapid expansion and cost effective nature of genotyping technology. Currently, the majority of traits utilized when estimating GEBV are measures occurring at a single time point or averaged across several time points. Alternatively, longitudinal models that describe the trajectory across time can be utilized to characterize the variation across animals across the time horizon for a specific trait. Models such as random regression or splines have been utilized in the past and are advantageous since they allow for the covariance between age classes (age (d) of an animal) to vary continuously across the trajectory [1–4]. While these models have seen widespread application with the use of pedigree data their use in conjunction with dense SNP panels, either for genomic prediction or trait architecture dissection is far less common. Previous research has utilized random regression models to characterize the effect of individual SNP across time using either simulated [5, 6] or real data [7] on a small number of SNPs. Characterizing SNP effects across a trajectory when the data is derived from dense SNP arrays (i.e. 1000+ SNPs) remains computationally demanding. In spite of this a genome wide association study (GWAS) using a longitudinal model offers several advantages, the main one being the ability to account for the heterogeneity of marker effects across time.

Growth and feed intake are economically important traits in swine production [8] and have been previously investigated using average daily gain and average daily feed intake, respectively [9, 10]. Complex traits such as growth and feed intake are often the result of dynamic systems. It is conceivable that different genes might play different roles along the growth and feed intake trajectory [11]. Longitudinal models offer the possibility to explicitly account for this heterogeneity. A recent GWAS was conducted by Tetens et al. [12] based on degressed EBV from a random regression model using Legendre polynomials for feed intake across specific phases of the lactation curve in dairy. To the authors’ knowledge no previous research has utilized the animal specific polynomial coefficients as a phenotype in a GWAS. The use of the polynomial coefficients could allow for the characterization of genes that impact specific components of the trajectory. Knowledge of these regions would in turn be advantageous to potentially identify genetic antagonisms involving the shape of the growth and feed intake trajectory.

Recently, a GWAS approach, referred to as single-step GWAS (**ssGWAS**), that utilizes all genotypes, phenotypes, and pedigree information jointly in one step has been proposed by Wang et al. [13] and validated using field data [14–16]. This approach allows for complex models such as random regression and multiple traits to be efficiently implemented. Furthermore, greater power and more precise estimates of variance components can be achieved by including non-genotyped animals if the number of genotyped animals is limited. The objective of this study is to perform a ssGWAS on the animal polynomial coefficients in order to identify genomic regions that impact specific polynomial coefficients of the growth and feed intake curves in Duroc boars.

## Results

### Genetic parameters

**Fig. 1:**
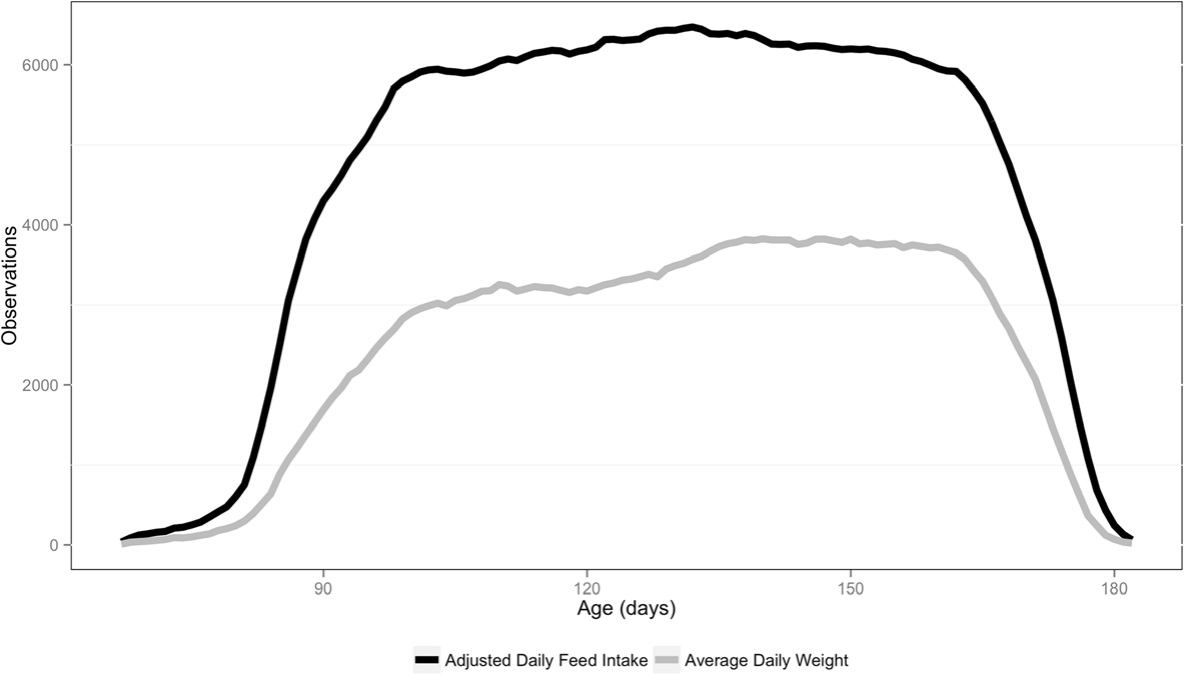
Observations by age for daily feed intake (DFI_Adj_) and average daily weight measurements (DBW_Avg_)

Corrected electronic FIRE (Feed Intake Recording Equipment, Osborne Industries, Inc., Osborne, KS, USA), daily feed intake (**DFI**_**Adj**_) and average daily weight measurements (**DBW**_**Avg**_) on 8981 (n = 525,240 observations) and 5643 (n = 283,607 observations) animals were utilized in a random regression analysis (order = 2). A blended relationship matrix (**H**) containing a SNP-derived genomic relationship matrix (**G**) and a pedigree numerator relationship matrix (**A**) was constructed to model the additive genetic relationship between animals [17]. The trajectory of each individual was split into three phases based on age classes. Phase 1, 2 and 3 included ages from 90 to 118d, 119 to 146d and 147 to 175d, respectively and the average heritability reported within each phase. Descriptive statistics and the number of observations within each class for both DFI_Adj_ and DBW_Avg_ are outlined in Table 1 and Fig. 1, respectively.

**Table 1:** Descriptive statistics for daily feed intake (DFI_Adj_) and average daily weight measurements (DBW_Avg_)

	DFI_Adj_	DBW_Avg_
Total Animals	8,981	5,643
Animals with Genotypes	858	590
Average Test Length (Min/Max), day	58.5 (20/98)	50.3 (20/95)
Average On-Test Age (Min/Max), day	97.0 (67/146)	100.2 (67/145)
Average Off-Test Age (Min/Max), day	162.9 (100/182)	164.2 (109/182)
Average Daily Feed Intake (± S.D), kg	2.03 (± 0.44)	-
Average Daily Gain (± S.D), kg	-	0.85 (± 0.22)

**Fig. 2:**
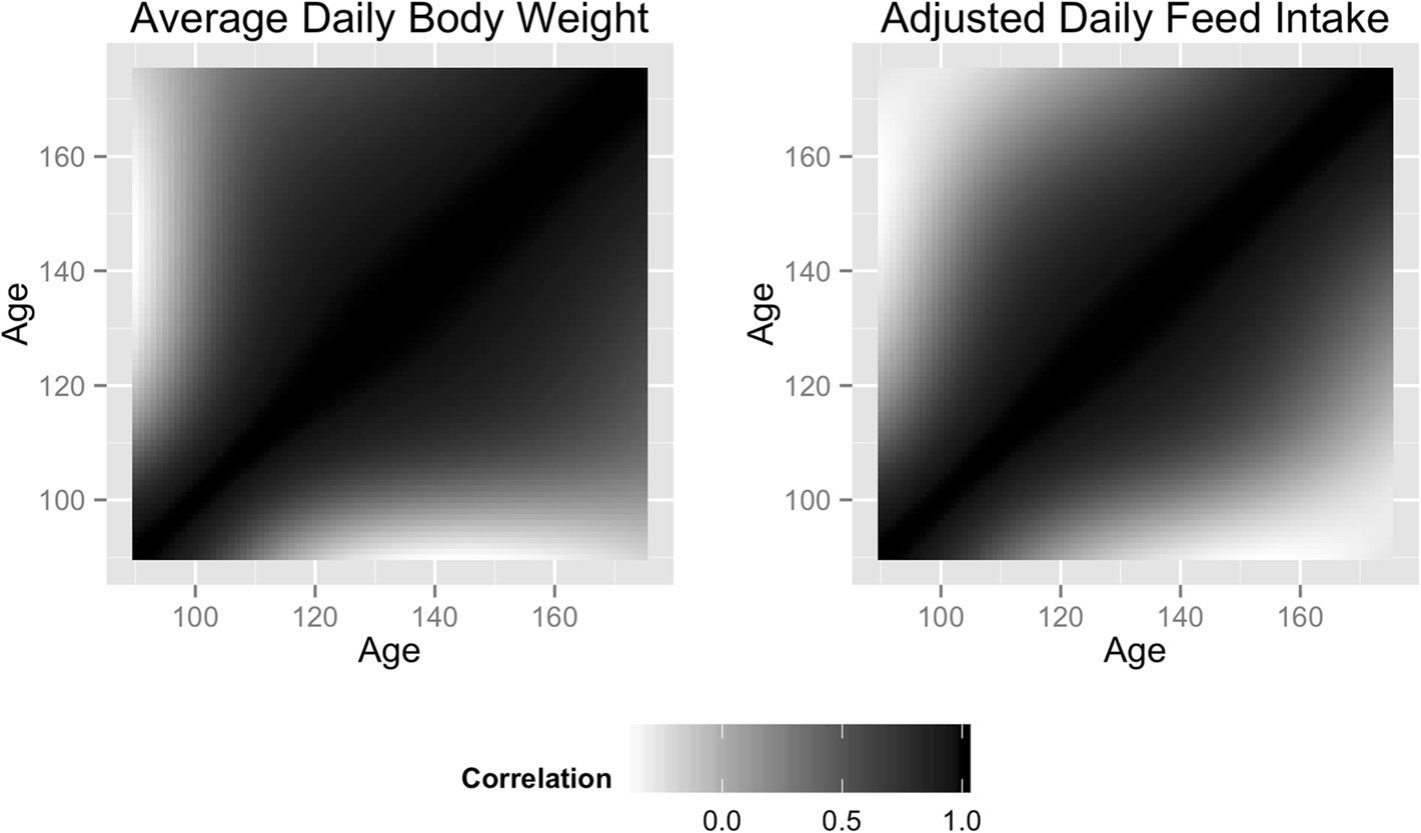
Genetic correlation across the trajectory for daily feed intake and average daily weight measurements

The estimated heritability for the traits ranged from 0.34 to 0.52 and 0.07 to 0.23 for DBW_Avg_ and DFI_Adj_ across the trajectory. Genetic correlations across the trajectory for DBW_Avg_ and DFI_Adj_ are depicted in Fig. 2. Correlations across age classes were large and positive for the majority of the trajectory for DBW_Avg_ (mean correlation: 0.75), although the correlation decreased slightly as the age classes grew further apart from each other. The mean correlation between phase 1 and 3 was 0.48. The genetic correlations across age classes for DFI_Adj_ (mean correlation: 0.54) were large and positive for age classes that were near each other. As the age distance increased, the correlation decreased with the lowest correlation found between age classes at the beginning and end of the trajectory. The mean correlation between phase 1 and 3 was 0.01. The average heritabilities for phase 1, 2 and 3 were 0.37, 0.45 and 0.50 for DBW_Avg_ and 0.08, 0.12 and 0.17 for DFI_Adj_. GEBV correlations and heritability within and across traits for each polynomial coefficient are outlined in Table 2. The correlation between the intercept and linear coefficient for DFI_Adj_ and DBW_Avg_ was moderate, while negligible between the intercept and quadratic coefficient. For both traits the correlation between the linear and quadratic coefficient was negative and moderate.

**Table 2:** Genomic estimated breeding value correlation (upper off-diagonal), average 10-SNP genomic estimated breeding value (lower off-diagonal) correlation and heritability (diagonal) estimates within and across polynomial coefficients for daily feed intake (DFI_Adj_) and average daily weight measurements (DBW_Avg_)

	Intercept DBW_Avg_	Linear DBW_Avg_	Quadratic DBW_Avg_	Intercept DFI_Adj_	Linear DFI_Adj_	Quadratic DFI_Adj_
Intercept DBW_Avg_	0.30	0.38	0.10	0.38	0.14	−0.21
Linear DBW_Avg_	0.37	0.18	−0.58	0.12	0.01	−0.09
Quadratic DBW_Avg_	0.04	−0.47	0.07	0.08	0.10	−0.09
Intercept DFI_Adj_	0.20	0.07	0.08	0.04	0.27	0.09
Linear DFI_Adj_	0.05	−0.02	0.10	0.28	0.07	−0.20
Quadratic DFI_Adj_	−0.13	−0.06	0.02	0.01	−0.27	0.04

### Genome-wide association study

A ssGWAS as described by Wang et al. [13] was conducted on the animal polynomial coefficients (i.e. intercept, linear and quadratic) for both DFI_Adj_ and DBW_Avg_. A total of 855 and 590 animals with both phenotypes and genotypes for DFI_Adj_ and DBW_Avg_ were used to conduct the association analysis on 31,366 autosomal SNP. The **G** part of the **H** matrix was utilized to iteratively estimate individual SNP effects from the animal specific GEBV for each polynomial coefficient. To characterize regions of the genome that had an impact on a given coefficient and to limit statistical noise and reduce the number of false positives 10-SNP sliding windows GEBV (WGEBV) was used. This was done to account for marker effects potentially being shared by adjacent SNP in high linkage-disequilibrium (LD). For each polynomial the significance level of the putative QTL window was estimated using a bootstrap analysis with 1000 replicates. Briefly, a bootstrap sample was generated for each observation by replacing the putative QTL windows with a sample from an independent standard normal distribution that was scaled by the residual variance from the full model. For each bootstrap sample the data was reanalyzed and the WGEBV re-computed. The p-value of a window was obtained based on the number of times a bootstrap sample WGEBV from the 1000 simulated exceeded the WGEBV from the real data. An arbitrary genome-wide significance value of P < 0.001 was adopted. Based on this, gene annotations for significant windows were obtained using the Biomart platform on Ensemble [18] through the ‘Biomart’ R package (http://www.bioconductor.org). To characterize the genetic relationship between polynomial coefficients within and across traits, the covariance between WGEBV across the genome for each trait polynomial combination was obtained. In addition, the WGEBV correlation averaged across the genome was compared to the GEBV correlation within and across traits.

Multiple regions were found to be significantly associated with specific polynomial coefficients based on the bootstrap analysis for both DFI_Adj_ and DBW_Avg_, as outlined in Table 3. Furthermore, the region on SSC9 was associated with both intercept terms for DFI_Adj_ and DBW_Avg_. Additional file 1: Figure S1 and Additional file 2: Figure S2 display the contribution of each WGEBV to the overall WGEBV variance for a given polynomial coefficient for DFI_Adj_ and DBW_Avg_, respectively. In general, the contribution of a particular region is heterogeneous across polynomial coefficients for both DFI_Adj_ and DBW_Avg_. The cumulative variance explained (number of windows) by significant windows for DFI_Adj_ was 1.0 (n = 10), 0.3 (n = 3) and 0.6 (n = 1) percent for the intercept, linear and quadratic polynomial coefficients, respectively. Similarly, cumulative variances explained (number of windows) by significant windows for DBW_Avg_ were 0.9 (n = 6), 0.2 (n = 1) and 0.5 (n = 5) percent for the intercept, linear and quadratic part of the trajectory, respectively. The WGEBV variance explained by significant regions for the trajectory was of 1.54 and 1.95 % for DBW_Avg_ and DFI_Adj_.

**Table 3:** QTL regions for the daily feed intake and average daily weight trajectory parameters

Trait	Coefficient	SSR^1^	Region^1^ (Start – End)	Reference SNP ID number	Location^1^ SNP with largest Impact^2^	Candidate gene (Gene Start - Stop)^1^	Function
Daily Feed Intake	Intercept	3	104.93 – 106.74	rs81374365	105,694,951		
		6	50.80 – 53.37	rs80971368	51,843,873	SIGLEC-5 (51.50 – 51.83)	Host Immune Response
		7	8.39 – 9.65	rs80858822	9,162,386	EDN1 (9.15 – 9.16)	Vasoconstriction & Kidney Functions
		8	30.60 – 31.03	rs81399022	30,934,915	TBC1D1 (31.01 – 31.05)	Energy Homoestasis
		9	8.78 – 9.38	rs331988332	8,950,525	UCP2 (9.15 – 9.16) UCP3 (9.17 – 9.18)	Energy Homoestasis
		9	65.35 – 66.81	rs81412363	66,001,271		
		9	146.99 – 147.55	rs81344419	147,145,126		
		11	78.00 – 78.81	rs81431902	78,262,842	TPP2 (78.24 – 78.32)	Anti-Satiety & Adipogenesis
		14	16.41 – 17.22	rs80800316	16,678,119	GLRA3 (16.70 – 16.78)	Behavior
		15	131.44 – 132.17	rs81454578	131,788,807	IGFBP5 (131.68 – 131.68)	Glucose Homeostasis
	Linear	2	124.53 – 125.09	rs81474570	124,815,799	CDO1 (124.82 – 124.83)	Cysteine homeostasis
		3	138.21 – 140.07	rs81336457	138,955,525		
		4	37.09 – 37.74	rs80849862	37,210,968	AZIN1 (37.09 – 37.74)	Polyamine Synthesis Regulation
	Quadratic	1	25.01 – 27.47	rs80803840	25,469,368	GPR126 (25.29 – 25.41)	Cell Signaling
Average Daily Body Weight	Intercept	1	176.19 – 177.76	rs80837663	176,186,716	PHLPP1 (176.12 – 176.23) MC4R (178.55 – 178.56)	Insulin Signaling Regulation of Metabolism
		4	44.24 – 46.14	rs80840184	44,687,188	NDUFAF6 (44.87 – 44.91)	Energy Homoestasis
		7	125.06 – 125.99	rs80927576	125,383,498	VRK1 (125.27 – 125.31)	Cell Growth & Division
		9	65.08 – 65.86	rs81412302	65,728,425		
		11	9.07 – 9.61	rs80786591	9,216,774		
		15	23.81 – 26.38	rs81451849	24,623,255		
	Linear	5	84.01 – 84.74	rs81325400	84,361,428	STAB2 (84.23 – 84.26)	Clearing of Metabolic Waste
	Quadratic	1	17.42 – 18.35	rs80807545	17,637,973		
		4	82.08 – 82.89	rs80787131	82,154,248	IMPAD1 (82.11 – 82.13)	Formation of Skeletal Elements
		6	100.55 – 106.32	rs81316981	100,548,492	CABLES1 (100.63 – 100.80)	Regulator of Cell Proliferation
		12	52.69 – 53.31	rs81327396	53,063,765		
		15	9.03 – 10.57	rs80840353	10,127,793	KYNU (9.03 – 10.57)	Tryptophan Metabolism

^1^Location of SNP in megabases based on swine genome build 10.2

^2^The impact of a particular SNP within a given regions was determine by calculating the SNP variance (2pq (SNP Effect)^2^)

The covariance between WGEBV polynomial coefficients across the genome is outlined in Additional file 3: Figure S3 and Additional file 4: Figure S4 for DFI_Adj_ and DBW_Avg_, respectively. In addition, the WGEBV correlation averaged across the genome is outlined in Table 2. Additional file 3: Figure S3 and Additional file 4: Figure S4 show how there are regions across the genome with a large degree of covariance across polynomial coefficients. In particular, a region on SSC9 (8.4-9.5) with a large and positive covariance between the intercept and quadratic coefficient for DBW_Avg_ was tagged as a putative QTL for both coefficients, although was not declared significant after the bootstrap analysis. The same region was declared significant for the intercept term for DFI_Adj_. Also, for DBW_Avg_ a region on SSC14 (15.6-17.2) had a positive covariance between the intercept and quadratic coefficient and the region was declared significant for the intercept term. Knowledge of regions that have a covariance that deviates from the average between two polynomial coefficients allows for the potential to alter genetic antagonisms regarding the shape of the trajectory through selection.

Genes within regions with a significant impact on the intercept coefficient for DFI_Adj_ were identified, involving energy homeostasis (*TBC1D1*, *UCP2, UCP3*), anti-satiety and adipogenesis (*TPP2*), behavior (*GLRA3*), glucose homeostasis (*IGFBP5*), host immune response and cell-to-cell interactions (*SIGLEC-5*), vasoconstriction and kidney function (*EDN1*). Furthermore, significant regions for higher order polynomial coefficients (i.e. linear and quadratic) included genes related to Cysteine homeostasis (*CDO1*), polyamine synthesis regulation (*AZIN1*) and cell signaling (*GPR126*). Regions that impacted the intercept coefficient for DBW_Avg_ included genes related to insulin signaling (*PHLPP1*), feeding behavior and regulation of metabolism (*MC4R*), energy homeostasis (*NDUFAF6*) and cell growth and division (*VRK1*). Similarly for linear and quadratic coefficients for DBW_Avg_ genes within significant regions were identified involved in the formation of skeletal elements (*IMPAD1*), the negative regulator of cell proliferation (CABLES1), clearing of metabolic waste (*STAB2*) and tryptophan metabolism (*KYNU*).

## Discussion

The heritability estimates derived from our study for DBW_Avg_ and DFI_Adj_ are in line with previous random regression estimates although genetic correlations between age classes are lower than previous studies. Utilizing FIRE systems, Haraldsen et al. [2] and Wetten et al. [3] estimated the heritability for the growth trajectory in Norwegian Duroc and Landrace boars using pedigree information. Estimates ranged between 0.32 to 0.35 and 0.17 to 0.25, respectively while genetic correlations across test days were never below 0.80. Using three weight measurements across the growth period Huisman et al. [4] estimated the heritability to range from 0.13 to 0.20 and the genetic correlation was the lowest (0.378) for measurements at the beginning and end of the growth phase. Zumbach et al. [19] using a population related to the one in the current study obtained heritability estimates of 0.04, 0.06 and 0.09 for daily, weekly, and bi-weekly intervals, respectively, using a repeatability model. Schnyder et al. [1] estimated the heritability for weekly mean daily feed intake from castrated Large White male pigs using pedigree information to range from 0.20 to 0.38 and the genetic correlation between weekly mean daily feed intake was large and positive with the lowest (r_g_ = 0.80) occurring for feed intake at week 1 and weeks at the end of the test period. Wetten et al. [3] estimated the heritability along the feed intake trajectory for Norwegian Duroc and Landrace boars using pedigree information to be from 0.09 to 0.11 for both breeds. The heritability for the polynomial coefficient for weekly mean feed intake was estimated by Schnyder et al. [1] and the majority of the variation was captured by the intercept (h^2^ = 0.32) with a smaller proportion captured by the linear (h^2^ = 0.06) and quadratic (h^2^ = 0.03) regression coefficients.

An alternative way to model growth curves using genomic information has been investigated by Silva et al. [20] using a nonlinear logistic regression model to estimate the regression functions for mature weight, start weight, and growth rate, and then used these as phenotypes in a GWAS. Silva et al. [20] estimated a moderately negative genetic correlation (r_g_ = −0.69) between mature weight and growth rate. This is in line with our results with moderately negative WGEBV and GEBV correlation between the linear and quadratic coefficients for DFI_Adj_ and DBW_Avg_. Although the method utilized by Silva et al. [20] for modeling growth curves does provide a way of obtaining mature weight breeding values, the ability to put different degrees of selection pressure across the trajectory and on specific polynomial coefficients is not possible. Random regression might allow to “bend the growth curve”. This has been investigated for example on lactation curves in dairy cattle using a restricted selection index in order to make cattle more persistent (i.e. reduced rate of decline in milk yield after peak milk yield) [21]. A different method could involve constructing a trait-specific marker derived relationship matrix as outlined by [22] that weights the genomic relationship matrix based on specific polynomials in order to place more emphasis on certain regions of the genome. Future research would need to verify the effectiveness of either approach for growth and feed intake in pigs.

A limited number of GWAS studies have investigated regions that impact feed intake and growth in pigs using average daily feed intake (ADFI) and average daily gain (ADG) as phenotypes [9, 10]. A common alternative metric to determine feed efficiency has been often utilized, referred to as residual feed intake (RFI) [9, 10, 23–26]. RFI is usually defined as the difference between the observed feed intake and the feed intake predicted based on production traits [27]. The limitations of using ADG, ADFI or RFI for a GWAS is that an animals feed intake and growth trajectory is not characterized and more importantly the gene effects are considered consistent across time. Due to a higher level of muscle deposition at the beginning of the trajectory compared to a higher level of fat deposition towards the end of the trajectory it is expected that different metabolic pathways are being differently regulated. A GWAS on the polynomial coefficient in a random regression model directly is advantageous because it allows for the additive genetic architecture to be understood for each polynomial coefficient. Furthermore, regions that have an effect across polynomial coefficients can be identified in order to characterize genetic antagonisms for the feed intake and growth trajectory. Longitudinal models could account for the fact that a gene effect might potentially not being consistent across time. It is expected that effects associated with the intercept coefficient would be homogenous across time while higher order polynomial coefficients, such as the linear and quadratic terms would capture transient effects across the trajectory. In the current study, a bootstrap analysis was conducted to declare significance based on WGEBV variance. A similar method has been utilized previously in GWAS studies [10, 28] and provides a robust, albeit computationally intensive way to conduct significant testing, when using the ssGWAS method.

In a previous study by Jiao et al. [10], a GWAS was conducted on ADG and ADFI using the same genetic line employed in the current work. A region located on SSC1 (166-170 Mb) was significantly associated with both ADG and ADFI. In the current study the same region was identified as a putative QTL for the ADFI intercept coefficient, but not ADG. The region did not pass the bootstrap significance threshold. A potential reason for the discrepancy is that the marker map we used was based on the 2^nd^ version of the SNP60k bead chip, whereas Jiao et al. [10] used the 1^st^ version. The marker map used in the current study is outlined in the supplementary attached marker map file (MarkerMap.xlsx). The linkage disequilibrium was investigated based on both genotypes used by Jiao et al. [10] and genotypes employed in the current study for SSC1 (168 – 180 Mb) and is illustrated in Additional file 5: Figure S5 and Additional file 6: Figure S6, respectively. As shown, there is strong LD between the region that Jiao et al. [10] found significant for ADG and ADFI and the MC4R gene based on the genotypes used in the current study, while LD was much weaker based on the genotypes used in the previous study. This could explain why the MC4R region instead of the region found by Jiao et al. [10] was found to be associated with DBW_Avg_ in the current study. Other reasons for the differences between the two analyses may be due to the fact that a Bayesian method was utilized in the previous study, a larger number of phenotyped and smaller number of genotyped individuals in the current dataset and different modeling techniques that allow for the covariance to change between age classes. A comparative analysis between Bayesian alphabet methods and ssGBLUP conducted by Wang et al. [15] highlighted how the strength and detection of associations depends on the methodology utilized and both have their advantages.

Multiple regions identified in the current study have been found to be previously associated with metrics related to feed intake and growth in both livestock species and humans. A region on SSC6 (50.8-53.4 Mb) was found to harbor the *SIGLEC-5* gene, which is contained within a large family of cell-surface transmembrane receptors that regulate host immune responses [29]. It has been found that *SIGLEC-5* weakly binds to leptin and potentially regulates leptin levels [30]. The region on SS7 (8.4-9.6 Mb) is in proximity of the *EDN1* (9.15 Mb) gene, a powerful endogenous vasoconstrictor peptide that is produced and released by the vascular endothelium [31]. A consistent body of literature in humans has shown how variants within this gene are associated with hypertension and obesity (see for example Tiret et al. [32]). A previous study by Onteru et al. [9] also found an association 2 Mb downstream of *EDN1*. The *TBC1D1* gene on SSC8 has been previously found to be associated with carcass traits in pigs [33]. The *TBC1D1* gene is a Rab-GTPase-activating related protein implicated in regulating the trafficking of glucose transporter 4 (*GLUT4*) storage vesicles to the cell surface in response to insulin and AMPK-activating stimuli in skeletal muscle [33]. A previous GWAS study for RFI by Do et al. [23] also found an association 2 Mb downstream of the *TBC1D1* gene. The two genes on SS9, *UCP2* and *UCP3*, produce carrier proteins of the inner membrane of the mitochondria that release protons in respiring mitochondria and expression of these enzymes is nutritionally and hormonally regulated and plays a role in the regulation of energy balance [34]. It has been shown in transgenic mice that overexpressing UCP2 and UCP3 result in decreased adiposity and increased hypothalamic NPY concentrations and feed intake [35]. The *TPP2* gene on SSC11 has been shown to have anti-satiety roles via the degradation of the satiety peptide cholecystokinin 8 and is required for mammalian adipogenesis [36]. A previous study by Gleason et al. [37] found that the absence of *IGFBP5* in mice results in an increase in size and mild glucose intolerance and is accentuated during diet-induced obesity. The region on SS1 that contained the gene (*GPR126*) was associated with the quadratic coefficient for DFI_Adj_ and has been previously found to be associated with human height [38] and weight gain in German Landrace boars [39]. Furthermore, a region 5 Mb upstream of *GPR126*, *PEX7* and *MAP3K5*, was found to be associated with RFI by Do et al. [26]. The *GPR126* gene is involved in cell signaling and has been shown to give rise to adolescent idiopathic scoliosis in humans, which is characterized by spinal deformations [40]. The progression of idiopathic scoliosis has been shown to be related to the growth and age of the individual therefore it is perhaps not surprising that the SNP effect would change across time in a non-linear manner based on functional analysis in humans [41].

Regions associated with the intercept coefficient for DBW_Avg_ was the gene *PHLPP1* on SSC1 which encodes a phosphatase that can terminate *Akt* signaling which in turn is able to regulate insulin levels. Andreozzi et al. [42] found that *PHLPP1* abundance is increased in adipose tissue and skeletal muscle of obese individuals, and is also significantly related to BMI and insulin resistance. A region 1.5 Mb upstream on SSC1, *MC4R*, has been previously found to be associated with ADFI and ADG [43]. Although the variant that has been shown to be associated with ADFI and ADG is not on the current chip, the region comprising *PHLPP1* and *MC4R* display high levels of linkage disequilibrium, as shown in Additional file 6: Figure S6, therefore it is possible either one or both of the genes are associated with the intercept coefficient for DBW_Avg_. The region on SSC6, which was associated with the quadratic coefficient for DBW_Avg_ contained the gene, *CABLE1*, which encodes a protein involved in cell cycle regulation by interacting with several cyclin-dependent kinases and has been previously found to be associated with height and menarche in humans [44]. The *STAB2* gene functions as a scavenger receptor to clear metabolic waste products from the circulation and in mice lacking the protein have been shown to display reduced hepatic clearance of waste products in the blood [45]. The region on SSC15 harbors the *KYNU* gene, which is involved in the kynurenine pathway, which is a major route for the majority of ingested tryptophan [46]. Tryptophan is the precursor of a wide array of metabolites, which are involved in a variety of aspects related to nutrition and metabolism [46].

## Conclusions

The incorporation of genomic information into random regression models has allowed for the identification of regions that are potentially associated with the shape of the growth and feed intake curve. These results have confirmed that the polynomial coefficients describing the individual’s growth and feed intake curve are impacted by different regions of the genome. Furthermore, the WGEBV covariance between growth and feed intake polynomial coefficients have been identified. Regions and genes with heterogeneous effects across time were identified by including linear and quadratic terms in the random regression model. Future research will involve using genomic information to modify the trajectory by constraining certain polynomials for both DBW_Avg_ and DFI_Adj_.

## Methods

### Data set

No animal care approval was required for the present manuscript because all records came from field data. Electronic FIRE (Feed Intake Recording Equipment, Osborne Industries, Inc., Osborne, KS, USA) feed intake and weight measurements on Duroc boars from June 23, 2004 to June 5, 2013 were initially utilized. Feed intake and weight observations were measured each time an animal visited the feeder. The pens measured 2.44 m by 5.61 m with an average of 1.4 squared meters per pig. Within each pen the fire stations measured 0.66 m by 1.70 m with a runway of 1.30 m. Detailed feed intake and body weight data editing steps are outlined in [47]. Briefly, feed intake editing techniques developed by Casey et al. [48] were used to identify and adjust for errors associated with feed intake observations. The editing procedures were: 1.) Identify and remove errors for each visit based on 16 criteria [48]; 2.) Sum error-free feed intake within each day for each pig; 3.) Estimate the effect of error counts on error-free feed intake by fitting a linear mixed model to error-free daily feed intake observations with the 16 error counts, contemporary group (concatenation of season, pen and year of birth), body weight on that day and ADG as covariates and pig as a random effect; 4.) Adjust error-free DFI_Adj_ for each pig and day for feed consumed during error visits based on estimates obtained from part 3. Lastly, animals with less than 20 DFI_adj_ observations were removed. The final number of DFI_Adj_ observations totaled 525,240 on 8981 animals.

Weight editing techniques developed by Zumbach et al. [19] were utilized to identify and remove errors. Briefly, utilizing robust regression with a bisquare weight function weight was fit to a quadratic regression of on-test day and linear regression of on-test age. Each data point from a robust regression procedure is assigned a weight (from 0 to 1) and weights that were less than 0.5 were treated as outliers and removed. Lastly, on-test ADG was computed by regressing weight on age and values less than .4 kg or greater than 2.0 kg were removed and the remaining weights were averaged by day (DBW_Avg_). The final number of DBW_Avg_ observations was 283,607 on 5643 animals. Descriptive statistics for DFI_Adj_ and DBW_Avg_ and the number of observations for each age (age (d) of an animal) is outlined in Table 1 and Fig. 1, respectively.

Genotypic data was derived from the Illumina PorcineSNP60K Bead (Illumina Inc., San Diego, CA; n = 3699) and the GGP-Porcine containing roughly 10,000 SNP (GeneSeek Inc., a Neogen Co., Lincoln, NE; n = 3621). Prior to the imputation of missing genotypes and imputation of low-density to medium-density, multiple quality control edits were conducted including removal of animals with call rates ≤ 0.90, SNP with call rates ≤ 0.90, SNP with a minor allele frequency (MAF) ≤ 0.02, and p-value < 0.0001 of a chi-square test for Hardy-Weinberg equilibrium. The Beagle software was used for imputation [49] and the mean (± SD) imputation accuracy (Beagle r^2^) across all SNP was 0.85 (± 0.15). The SNP unmapped to the swine genome build 10.2 and SNP on sexual chromosomes were also excluded from the analysis. Furthermore, the map file used in the current analysis was based on version 2 of the Illumina PorcineSNP60K Bead genotype platform and any markers that were not in common were removed. Only animals with both phenotypes and genotypes were used in the analysis and totaled 858 and 590 for DFI_Adj_ and DBW_Avg_, respectively. Animals were derived from both the medium-density (DFI_Adj_: n = 786; DBW_Avg_: n = 587) and the low-density panel (DFI_Adj_: n = 70; DBW_Avg_: n = 3). Prior to analysis the MAF for the genotyped animals used in the analysis was checked and SNP with a MAF < 0.002 were removed, resulting in 31,366 SNP utilized in the analysis.

### Statistical analysis

Legendre polynomials (order = 2) were used to model the trajectory of DFI_Adj_ and DBW_Avg_. Prior to the analysis, age was standardized to have values from −1 to 1 to ensure numerical stability. Variance components were estimated by REML using the REMLF90 software [50]. A homogenous variance structure was utilized to decrease model complexity and similar results were found when a heterogeneous residual variance structure was utilized (data not shown). The model for DFI_Adj_ and DBW_Avg_ was,

$$ {y}_{ijkmn} = \mu + C{G}_i+ Parit{y}_j + {\displaystyle {\sum}_{k=0}^2}{\varphi}_{mnk}{\beta}_k+{\displaystyle {\sum}_{k=0}^2}{\varphi}_{mnk}{u}_{mk}+{\displaystyle {\sum}_{\mathrm{k}=0}^2}{\varphi}_{mnk}p{e}_{mk}+{e}_{ijkmn} $$

where y_ijkmn_ was DFI_Adj_ or DBW_Avg_, μ was the average DFI_Adj_ or DBW_Avg_, CG_i_ was the fixed effect of contemporary group (concatenation of birth year, season and pen), Parity_j_ was the fixed effect of parity of the dam (1,2,3+), β_k_ was the fixed regression coefficient of age, u_mk_ was the k^th^ random regression for animal_m_, pe_mk_ was the k^th^ random regression for the permanent environmental effect of animal_m_ and e_ijkmn_ was the residual. The effect φ_mnk_ was the k^th^ Legendre polynomial for animal_m_ at age_n_. It was assumed **u** ~ *N*(0, **H** ⊗ **G**), where **G** was a 3x3 (co)variance matrix for the animal Legendre polynomials and **pe** ~ *N*(0, **I** ⊗ **P**), where **P** was a 3x3 (co)variance matrix for animal permanent environmental Legendre polynomials. Construction of the **H** matrix consisted of blending a 3-generation pedigree derived numerator relationship matrix and a SNP-derived genomic relationship matrix with a weighting factor of 0.995 and 0.005, respectively [17]:

$$ {\mathbf{H}}^{\hbox{-} 1} = {\mathbf{A}}^{-1}+\left[\begin{array}{cc}\hfill 0\hfill & \hfill 0\hfill \\ {}\hfill 0\hfill & \hfill {\boldsymbol{G}}^{-1}\ \hbox{--} {\boldsymbol{A}}_{22}^{-1}\hfill \end{array}\right] $$

where **A**_**22**_ is a numerator relationship matrix for genotyped animals. The genomic relationship matrix (**G**) was created by weighting each marker contribution by its expected variance:

$$ \boldsymbol{G}=\boldsymbol{Z}\boldsymbol{D}\boldsymbol{Z}\hbox{'}, $$

where **Z** is a matrix of gene content containing genotype (−1, 0, 1) adjusted for allele frequencies and **D** is a diagonal matrix with elements containing the reciprocal of the expected marker variance [49]. In order to determine the change in heritability and genetic correlation across time, the trajectory was split into three phases. Only age classes from 90 to 175d were used when calculating the heritability within a phase and the genetic correlation across phases. This was done due to large variance component standard errors from a small sample size at the beginning and end of the trajectory. Phase 1, 2 and 3 consisted of age classes 90 to 118d, 119 to 146d and 147 to 175d, respectively.

### Genome-wide association mapping

A single step genome-wide association study (ssGWAS) as described by Wang et al. [13] was conducted on the animal specific polynomial coefficients (i.e. intercept, linear and quadratic) for both DFI_Adj_ and DBW_Avg_. Briefly, the GEBV solutions from the previous analysis were used to estimate marker effects through an iterative process. In the first round the GEBV solutions are utilized to estimate marker effects based on a **G** matrix weighted by the expected marker variance [51]. In successive iterations marker effects are then recalculated with a similar process but with SNP expected variance in **G** replaced by the realized variance obtained in the previous iteration. The reweighting process effectively increases the weight of SNP with large effect and decreases those with small effects. A detailed description of the iterative algorithm is outlined in Wang et al. [13] under the ‘Scenario 1’ procedure. In our study the reweighting process was repeated twice to ensure stability of the marker effects estimates [15].

Similar to Sun et al. [14, 16, 52], a 10-SNP sliding window approach was utilized to characterize regions that have a large effect on a specific parameter of the trajectory and to declare significance for these regions using bootstrap methods. This was done to account for marker effects potentially being shared by adjacent SNP in high linkage-disequilibrium (LD) and to remove assumptions regarding the start and stop site of a region in LD with a QTL. Furthermore, it has been shown that SNP segments are useful to discriminate important effects from statistical noise [52] and it has been shown by Beissinnger et al. [53] that either 5 or 10 SNP window sizes had the most favorable ratio of detection rate to false-positive rate. The variance of 10-SNP sliding windows GEBV (WGEBV) was computed for each individual by multiplying the estimated SNP effects with their respective genotypes and summing across all SNP within the window. The WGEBV variance was then used in a two-stage approach to identify regions with large effects. The first-stage involved isolating regions with large effects by keeping the top 5 % WGEBV regions. The second stage involved sorting the windows by chromosome and genome location. Overlapping WGEBV were aggregated into one region and the aggregated regions were ranked based on their maximum WGEBV variance. The top 10 % aggregated regions were tagged as putative QTL (n = 17) to be further investigated for significance.

### Declaring significance

Within each trait and polynomial coefficient the significance of putative QTL regions were determined based on a bootstrap analysis with 1000 replicates. Bootstrap samples were constructed using the estimated marker effects across the 3 polynomial coefficients to construct the distribution of the test statistic (WGEBV variance) for each putative QTL window within each polynomial coefficient. A bootstrap sample was constructed according to the null hypothesis of no QTL in the identified SNP window [28]. A bootstrap sample of vector y for replicate k (y_(k)_) was constructed from the estimated fixed effects, random permanent environmental effects, SNP effects across all three polynomials, excluding SNP contained within the putative QTL and adding a simulated residual for each animal and day combination. The simulated residual was generated from sampling an independent standard normal distribution that was scaled by the residual variance from the full model. Using the predicted phenotype generated from the full model for animal_i_ on day_j_, a bootstrap sample for replicate_k_ was generated by:

$$ {\tilde{y}}_{ij(k)} = {\widehat{y}}_{ij(k)}\hbox{-}\ {\widehat{u}}_{ij(k)}+{\tilde{u}}_{ij(k)}+{e}_{ij(k)}, $$

where *ỹ*_*ij*(*k*)_ refers to the bootstrap sample phenotype, *ŷ*_*ij*(*k*)_ refers to the predicted phenotype from the full analysis, *û*_*ij*(*k*)_ refers to the GEBV from the full analysis, *ũ*_*ij*(*k*)_ refers to the GEBV with SNP contained within the putative QTL window excluded for a given polynomial coefficient and *e*_*ij*(*k*)_ refers to a simulated residual.

For each bootstrap sample the ssGWAS reweighting procedure was conducted and the resulting marker effects were again partitioned into sliding windows and WGEBV were obtained as described above. The WGEBV for each putative QTL window was accumulated across all bootstrap samples and compared to the WGEBV variance test statistic derived from the real data. The p-value of a window was reported as the number of times a bootstrap statistic from the 1000 simulated exceeded the test statistic from the real data. Significance was declared using an empirical cutoff of P < 0.001 (i.e. test static from real data was never greater than any bootstrap statistic). Furthermore, the imputation accuracy (Beagle r^2^) for all significant SNPs within a region were checked to ensure no spurious results. The percent of the additive genetic variation explained by all significant QTL regions for each polynomial coefficient was calculated using the following formula:

$$ \mathrm{Genetic}\ \mathrm{Variation}\ \mathrm{Explained}:\ \frac{{\displaystyle {\sum}_1^r}\left(Var{\displaystyle {\sum}_{i=1}^{i={r}_i}}{m}_i{\hat{u}}_i\right)}{Var{\displaystyle {\sum}_1^m}{m}_i{\hat{u}}_i} \times 100 $$

Where *r* refers to the region, **m**_**i**_ is a vector of genotypes for SNP *i* for all individuals, and *û*_*i*_ is the effect of SNP *i*. Gene annotations for significant windows were obtained using the Biomart platform on Ensemble [18] through the ‘Biomart’ R package (http://www.bioconductor.org).

## Additional files

Additional file 1: Figure S1. Contribution of each 10-SNP sliding window GEBV variance to the overall variance for a given polynomial coefficient for average daily weight measurements. Additional file 2: Figure S2. Contribution of each 10-SNP sliding window GEBV variance to the overall variance for a given polynomial coefficient for daily feed intake. Additional file 3: Figure S3. 10-SNP sliding window GEBV covariance across the genome for average daily weight. Additional file 4: Figure S4. 10-SNP sliding window GEBV covariance across the genome for daily feed intake. Additional file 5: Figure S5. Linkage disequilibrium based on r^2^ scores^1^ of the region on SSC1 from 165 to 180 Mb based on the genotypes and their location used by Jiao et al. [10]. The red arrow refers to the SNP (ALGA0006684) that was associated with ADG and ADFI in Jiao et al. [10]. The black and blue arrow refer to the SNP that is associated with the intercept parameter for average daily weight measurements and the SNP that is closest to MC4R gene, respectively. ^1^ *r* ^2^ scores: white squares, *r* ^2^ = 0; black squares, *r* ^2^ = 1; grey squares, 0 < *r* ^2^ < 1. Additional file 6: Figure S6. Linkage disequilibrium based on r^2^ scores^1^ of the region on SSC1 from 165 to 180 Mb based on the genotypes used in the current study. The red arrow refers to the SNP (ALGA0006684) that was associated with ADG and ADFI in Jiao et al. [10]. The black and blue arrow refer to the SNP that is associated with the intercept parameter for average daily weight measurements and the SNP that is closest to MC4R gene, respectively. ^1^ *r* ^2^ scores: white squares, *r* ^2^ = 0; black squares, *r* ^2^ = 1; grey squares, 0 < *r* ^2^ < 1.
