# Supplementary figures and images for: Genome-wide association study on legendre random regression coefficients for the growth and feed intake trajectory on Duroc Boars
Source: BMC Genet. 2015 May 30;16:59. doi: 10.1186/s12863-015-0218-8 (PMC4449572; doi:10.1186/s12863-015-0218-8)

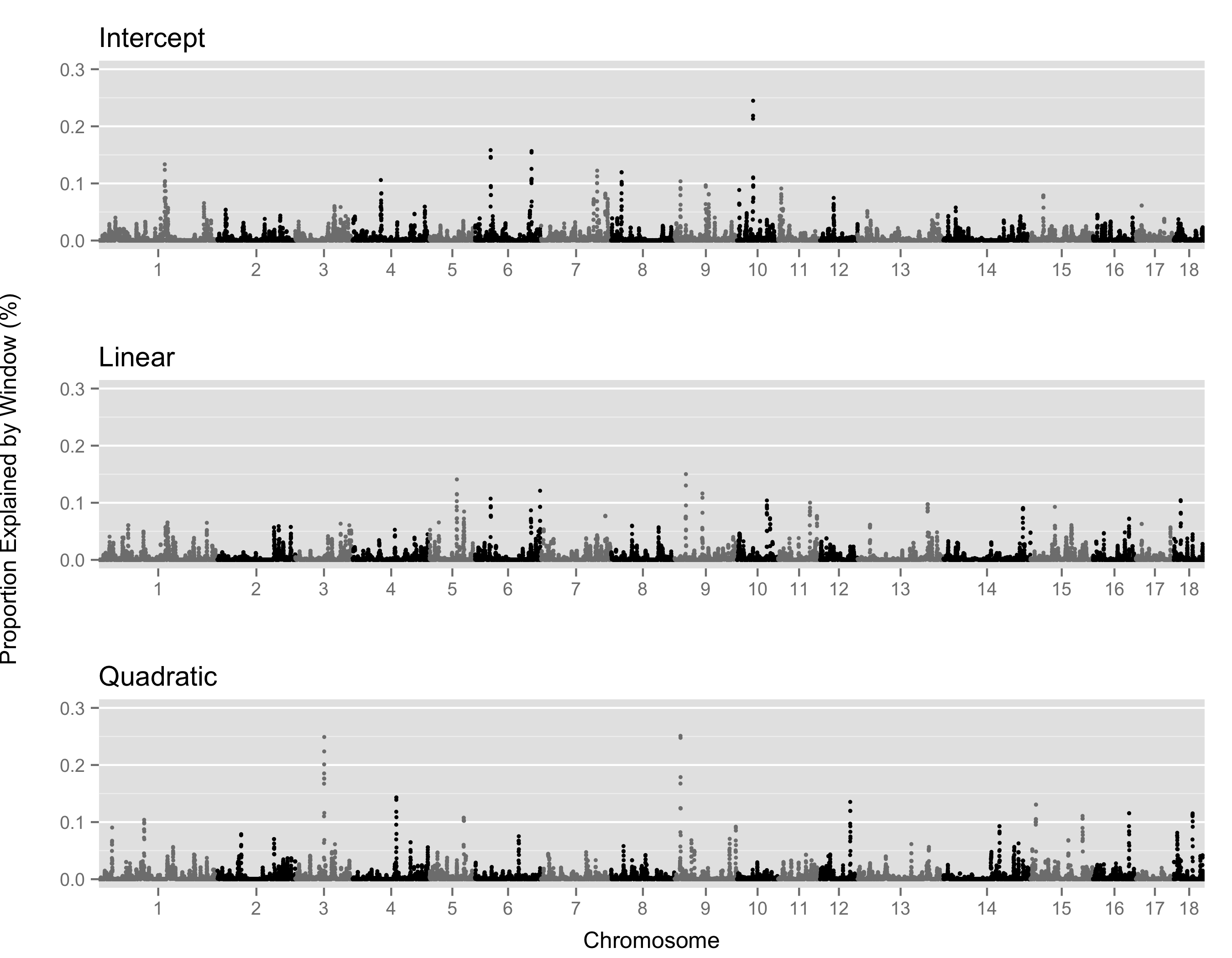

Supplement: Additional file 1: Figure S1. — Contribution of each 10-SNP sliding window GEBV variance to the overall variance for a given polynomial coefficient for average daily weight measurements. [file 12863_2015_218_MOESM1_ESM.png]

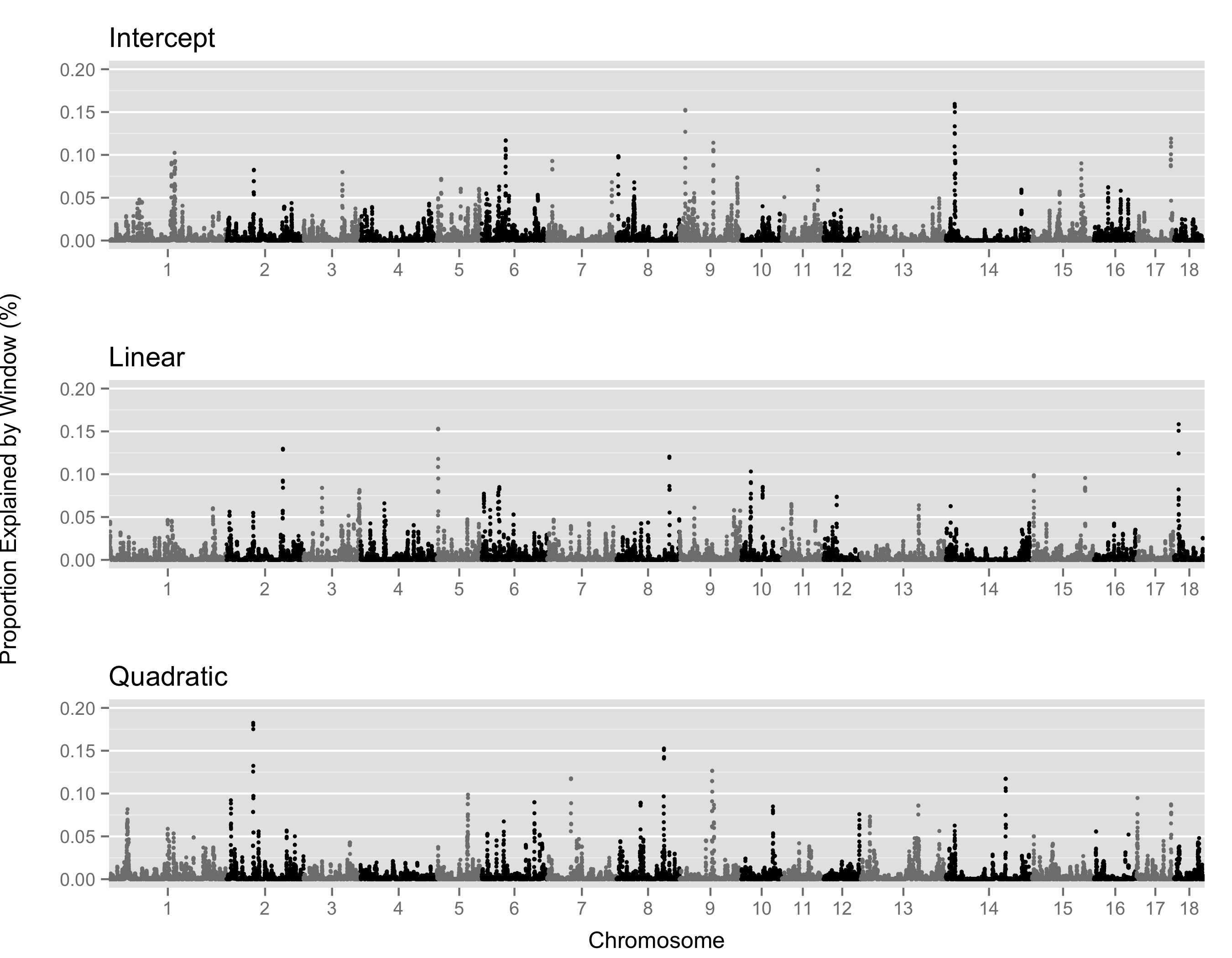

Supplement: Additional file 2: Figure S2. — Contribution of each 10-SNP sliding window GEBV variance to the overall variance for a given polynomial coefficient for daily feed intake. [file 12863_2015_218_MOESM2_ESM.png]

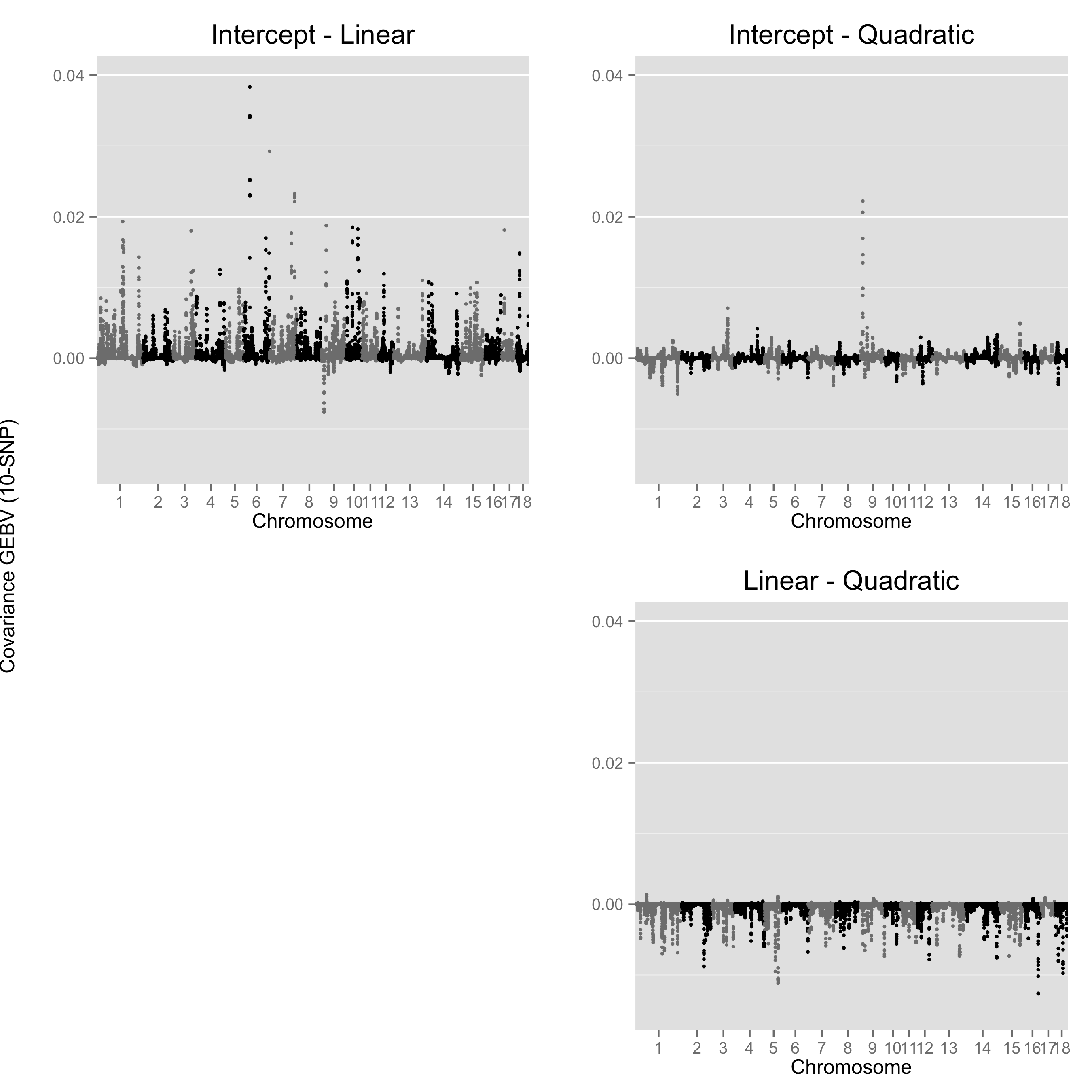

Supplement: Additional file 3: Figure S3. — 10-SNP sliding window GEBV covariance across the genome for average daily weight. [file 12863_2015_218_MOESM3_ESM.png]

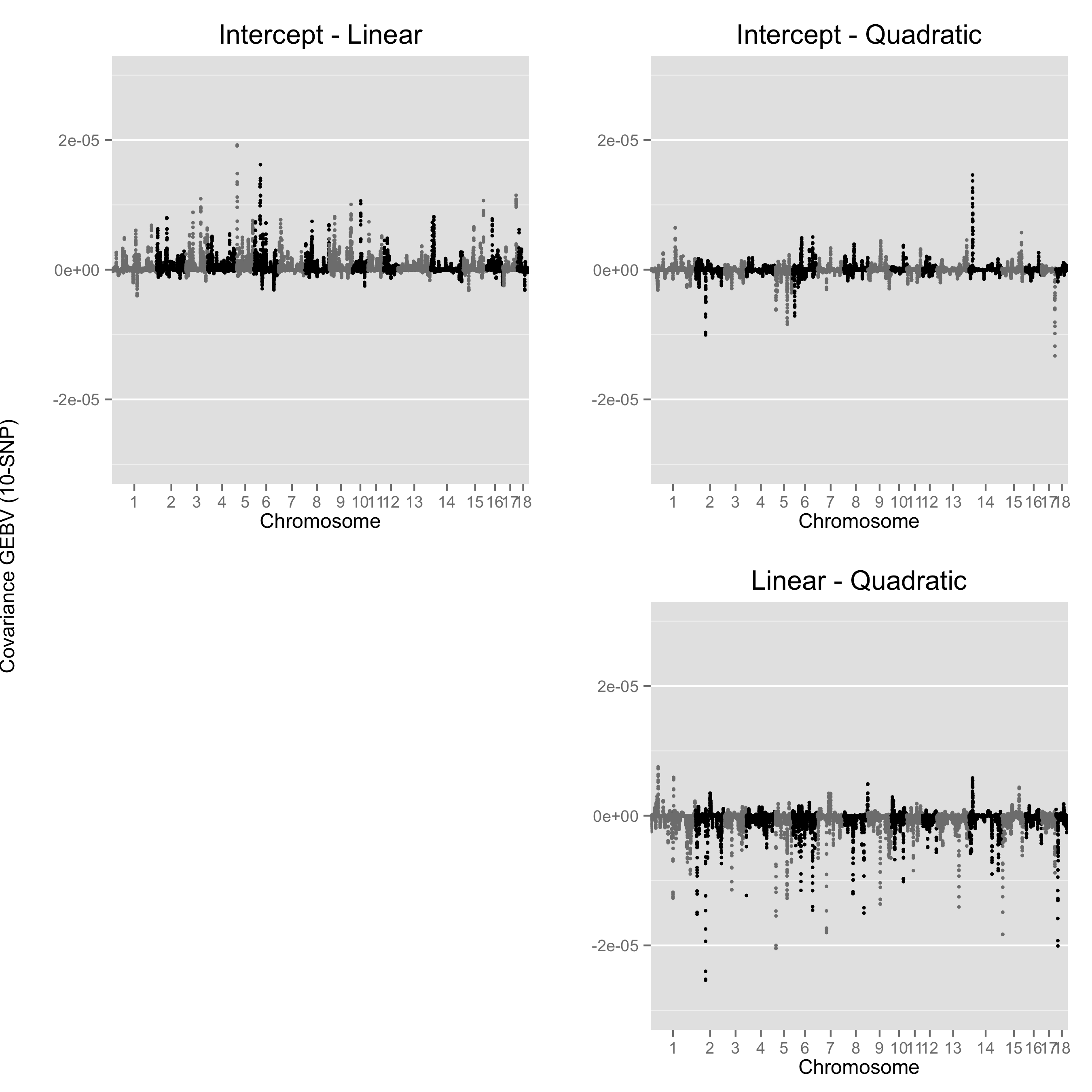

Supplement: Additional file 4: Figure S4. — 10-SNP sliding window GEBV covariance across the genome for daily feed intake. [file 12863_2015_218_MOESM4_ESM.png]

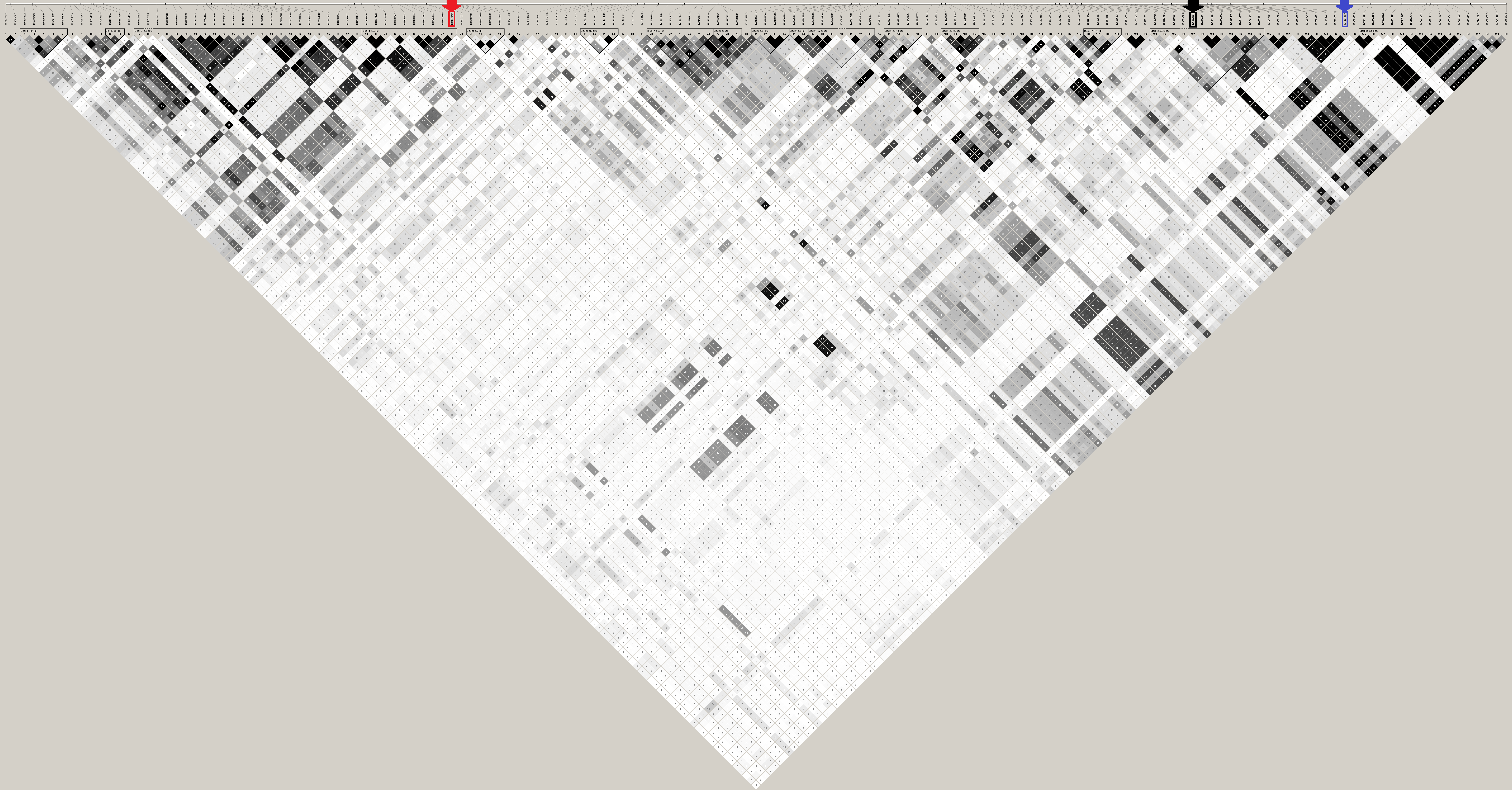

Supplement: Additional file 5: Figure S5. — Linkage disequilibrium based on r2 scores1 of the region on SSC1 from 165 to 180 Mb based on the genotypes and their location used by Jiao et al. [10]. The red arrow refers to the SNP (ALGA0006684) that was associated with ADG and ADFI in Jiao et al. [10]. The black and blue arrow refer to the SNP that is associated with the intercept parameter for average daily weight measurements and the SNP that is closest to MC4R gene, respectively. 1 r 2 scores: white squares, r 2 = 0; black squares, r 2 = 1; grey squares, 0 < r 2 < 1. [file 12863_2015_218_MOESM5_ESM.png]

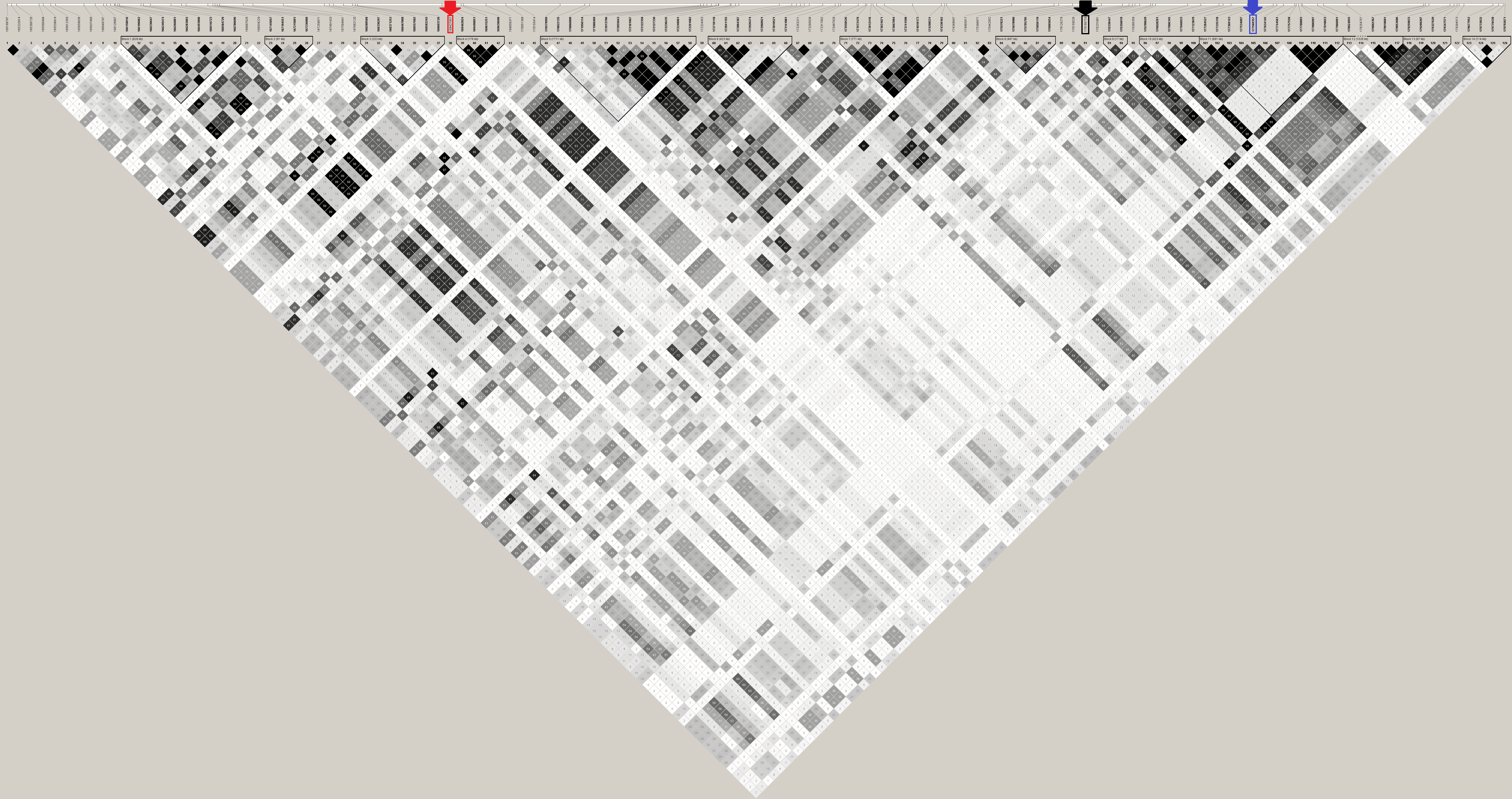

Supplement: Additional file 6: Figure S6. — Linkage disequilibrium based on r2 scores1 of the region on SSC1 from 165 to 180 Mb based on the genotypes used in the current study. The red arrow refers to the SNP (ALGA0006684) that was associated with ADG and ADFI in Jiao et al. [10]. The black and blue arrow refer to the SNP that is associated with the intercept parameter for average daily weight measurements and the SNP that is closest to MC4R gene, respectively. 1 r 2 scores: white squares, r 2 = 0; black squares, r 2 = 1; grey squares, 0 < r 2 < 1. [file 12863_2015_218_MOESM6_ESM.png]
